# Current Approaches to Vaccine Safety Using Observational Data: A Rationale for the EUMAEUS (Evaluating Use of Methods for Adverse Events Under Surveillance-for Vaccines) Study Design 

**DOI:** 10.3389/fphar.2022.837632

**Published:** 2022-03-22

**Authors:** Lana YH Lai, Faaizah Arshad, Carlos Areia, Thamir M. Alshammari, Heba Alghoul, Paula Casajust, Xintong Li, Dalia Dawoud, Fredrik Nyberg, Nicole Pratt, George Hripcsak, Marc A. Suchard, Dani Prieto-Alhambra, Patrick Ryan, Martijn J. Schuemie

**Affiliations:** ^1^ Division of Informatics, Imaging and Data Sciences, University of Manchester, Manchester, United Kingdom; ^2^ Department of Biostatistics, University of California, Los Angeles, Los Angeles, CA, United States; ^3^ Nuffield Department of Clinical Neurosciences, University of Oxford, Oxford, United Kingdom; ^4^ Medication Safety Research Chair, King Saud University, Riyadh, Saudi Arabia; ^5^ Faculty of Medicine, Islamic University of Gaza, Gaza, Palestine; ^6^ Real-World Evidence, Trial Form Support, Barcelona, Spain; ^7^ Centre for Statistics in Medicine, NDORMS, University of Oxford, Oxford, United Kingdom; ^8^ Faculty of Pharmacy, Cairo University, Giza, Egypt; ^9^ School of Public Health and Community Medicine, Institute of Medicine, Sahlgrenska Academy, University of Gothenburg, Gothenburg, Sweden; ^10^ Clinical and Health Sciences, University of South Australia, Adelaide, SA, Australia; ^11^ Department of Biomedical Informatics, Columbia University, New York, NY, United States; ^12^ Department of Human Genetics, University of California, Los Angeles, Los Angeles, CA, United States; ^13^ Health Data Sciences, Medical Informatics, Erasmus Medical Center University, Rotterdam, Netherlands; ^14^ Observational Health Data Analytics, Janssen R&D, Titusville, NJ, United States

**Keywords:** vaccine safety surveillance, methods evaluation, real-world data, study design, bias

## Abstract

Post-marketing vaccine safety surveillance aims to detect adverse events following immunization in a population. Whether certain methods of surveillance are more precise and unbiased in generating safety signals is unclear. Here, we synthesized information from existing literature to provide an overview of the strengths, weaknesses, and clinical applications of epidemiologic and analytical methods used in vaccine monitoring, focusing on cohort, case-control and self-controlled designs. These designs are proposed to be evaluated in the EUMAEUS (Evaluating Use of Methods for Adverse Event Under Surveillance–for vaccines) study because of their widespread use and potential utility. Over the past decades, there have been an increasing number of epidemiological study designs used for vaccine safety surveillance. While traditional cohort and case-control study designs remain widely used, newer, novel designs such as the self-controlled case series and self-controlled risk intervals have been developed. Each study design comes with its strengths and limitations, and the most appropriate study design will depend on availability of resources, access to records, number and distribution of cases, and availability of population coverage data. Several assumptions have to be made while using the various study designs, and while the goal is to mitigate any biases, violations of these assumptions are often still present to varying degrees. In our review, we discussed some of the potential biases (i.e., selection bias, misclassification bias and confounding bias), and ways to mitigate them. While the types of epidemiological study designs are well established, a comprehensive comparison of the analytical aspects (including method evaluation and performance metrics) of these study designs are relatively less well studied. We summarized the literature, reporting on two simulation studies, which compared the detection time, empirical power, error rate and risk estimate bias across the above-mentioned study designs. While these simulation studies provided insights on the analytic performance of each of the study designs, its applicability to real-world data remains unclear. To bridge that gap, we provided the rationale of the EUMAEUS study, with a brief description of the study design; and how the use of real-world multi-database networks can provide insights into better methods evaluation and vaccine safety surveillance.

## Introduction

Ever since coronavirus disease 2019 (COVID-19) was first reported in Wuhan, over 263 million cases and 5.2 million deaths have been reported worldwide (John HopkinsUniversity, 2021). While good hygiene and public health measures have been fundamental weapons against COVID-19, developing a preventative vaccine is critical to decreasing spread and potentially ending the pandemic. Therefore, researchers embarked on an unprecedented global effort to produce several vaccines in record time, advancing from preclinical studies to emergency use approval within 1 year (Ball, 2021). As of 01 December 2021, over eight billion vaccine doses had been administered globally (Holder, 2021). At least 26 COVID-19 vaccines have been approved by at least one country (Basta, 2020), four of which were approved by the European Medicines Agency (EMA) (European Medicines Agency EMA, 2021), and three by the United States Food and Drug Administration (US FDA) (US Food and Drug Administration FDA, 2021).

Although vaccine safety has been rigorously monitored in clinical trials, rare adverse events often go undetected as trial participants are often limited in number and followed for a relatively short duration under controlled circumstances. For example, major thromboembolic events and thrombocytopenia following the AstraZeneca-Oxford (Vaxzevria) vaccine were only detected when used in a larger population outside the clinical trial setting (European Medicines Agency EMA, 2020a). Recent data from the United Kingdom suggests an incidence of 20.7 per million doses in those aged 18–49 years compared to 10.8 per million doses in those 50 years and older (UK Medicines and Healthcare products Regulatory Agency, 2021). Other adverse events may also go undetected due to the exclusion of certain subpopulations (e.g., pregnant women or some age groups) in clinical trials. Thus, efficient routine post-marketing safety surveillance is increasingly crucial to provide accumulating real-world evidence, especially when vaccination coverage is expected to be high and vaccination roll-out is rapid (European Medicine Agency EMA, 2020b).

In the United States, the Vaccine Adverse Event Reporting System (VAERS), run by the FDA and the Centers for Disease Control and Prevention (CDC), is widely used to identify known and potentially new adverse events following immunization (AEFI) in a population (Moro et al., 2016; Centers for Disease Control and Prevention Vaers, 2021). Another well-established system is the Vaccine Safety Datalink (VSD), a collaborative project between the CDC and nine health care organizations, that uses electronic health records (EHRs) and administrative claims data to monitor vaccine safety and study rare and serious AEFI (Centers for Disease Control and Prevention VSD, 2021). Additionally, the Clinical Immunization Safety Assessment (CISA) network, a partnership between the CDC and six academic centres with vaccine safety expertise, works to improve understanding of AEFI at the individual patient-level (Centers for Disease Control and Prevention Cisa, 2021). The Post-licensure Rapid Immunization Safety Monitoring System (PRISM), which is part of the FDA’s Sentinel Initiative, focuses on vaccine safety using health insurance claims to identify and evaluate possible safety issues relating to licensed vaccines (Baker et al., 2013).

In Europe, the following monitoring options for COVID-19 vaccines have been proposed by the EMA: 1) periodic safety reports; 2) collection of exposure data (including observed-to-expected analyses); 3) observational research in collaboration with academic and private partners; and 4) spontaneous reporting of suspected adverse reactions (European Medicine Agency EMA, 2020b).

The World Health Organization recently published a safety surveillance manual specifically for COVID-19 vaccines, of which four categories of surveillance strategies were identified, including the following (World Health Organization (WHO), 2021):

1. Passive surveillance–when an AEFI occurs, only then reports are generated and a network is notified through surveillance sites. This includes spontaneous self-reporting by patients.
2. Active surveillance–a standard protocol is in place to help health-care professionals review medical records and identify suspected cases of AEFI.
3. Cohort event monitoring–health-care professionals are trained to conduct follow-up on those who have been vaccinated through defined channels such as phone-calls, home visits, email etc.
4. Sentinel surveillance–AESI data is collected only from a limited network of carefully selected reporting sites.

Since the aim of this review is to provide the rationale of the EUMAEUS study design, we will therefore be only focusing on current approaches employed to monitor vaccine safety, using observational real-world data. The availability of real-world data is increasingly being recognised as an important useful complementary data source to monitor for AEFI signals in real time (Leite et al., 2016). The Observational Health Data Sciences and Informatics (OHDSI) community is a global initiative that converts clinical data from EHRs, claims and registries into the Observational Medical Outcomes Partnership (OMOP) common data model (CDM). This standardization of data has allowed researchers to conduct large-scale patient-level prediction studies (Centers for Disease Control and Prevention Cisa, 2021), perform electronic phenotyping (Baker et al., 2013), and characterize diseases, including newer diseases such as COVID-19 (e.g., CHARYBDIS: Characterizing Health Associated Risks, and Your Baseline Disease In SARS-CoV-2) (Observational Health Data Sciences and Informatics (OHDSI), 2021; Morales et al., 2021). OHDSI’s contribution to the scientific community during this pandemic has included an early study on the safety profile of hydroxychloroquine, which received attention from the FDA and EMA in mid-2020 (Lane et al., 2020), and a more recently published study on the use of repurposed drugs and adjunctive treatments in COVID-19 involving >300,000 patients spanning across three continents (Prats-Uribe et al., 2021). In the next phase of COVID-19 related research, OHDSI is focusing on vaccine safety surveillance (VSS) - EUMAEUS (Evaluating Use of Methods for Adverse Event Under Surveillance–for vaccines), which aims to evaluate the performance of methods across various study designs to identify vaccine safety signals in a real-world setting. Here, we provide an overview of the previous knowledge regarding strengths, weaknesses, and clinical applications of epidemiologic and analytical methods used in vaccine monitoring that were selected for evaluation in EUMAEUS, with a brief rationale and overview of the EUMAEUS study design at the end of this review.

### Review of Epidemiological Designs for Vaccine Safety Surveillance

In the following sections, we review the various study designs commonly used in VSS in detail, including strengths, weaknesses, clinical applications, as well as some of the common types of confounding and biases associated with these study designs. We conclude this section of the review by discussing the strengths and limitations of using real-world data for methods evaluation and VSS.

### Types of Common Study Designs in Vaccine Safety Surveillance

One of the critical aspects of vaccine surveillance is determining whether the rate of an adverse event following immunization is greater than would have occurred by chance alone (i.e., without the immunization). To do this a comparator population and/or time is required to determine the baseline rate of disease. In epidemiological studies, the comparator may be derived from other, non-vaccinated patients (cohort studies), or from periods of time when the same individual was not-vaccinated (self-controlled designs). Here we describe the details of the most common study designs (i.e., cohort, case-control, self-controlled case series (SCCS), and self-controlled risk intervals (SCRI)) and their clinical applications described in literature. Table 1 gives an overview of each study design, including their advantages, disadvantages and clinical applications.

**TABLE 1:** Overview of Study Designs

Study Design	Description	Advantages	Disadvantages	Clinical Applications
Historical Cohort	Comparison between observed incidence of adverse events vs. expected incidence based on historical data.	Greater statistical power to detect rare adverse events; Improved timeliness in signal detection.	Subject to temporal confounders, changing trends in detection of adverse events and variation in diagnostic/coding criteria over time.	Pediatric vaccines; Tdap vaccine; HPV vaccine; H1N1 vaccine.
Cohort	Comparison of incidence ratio of adverse events between vaccinated vs. unvaccinated population.	Easy to implement-abundant data available; Use matching/stratification to control for confounders.	Confounding by indication/unmeasured confounders; Susceptible to misclassification of exposure.	Intussusception and rotavirus vaccine; Autism spectrum disorders and various vaccines.
Case-control	Comparison of cases vs. noncases from the same source population from the same time-period.	Uses small data sample from entire cohort, cost efficient; Uses matching to control for time-varying confounders.	Confounding by indication/unmeasured confounders; Selection bias; Susceptible to misclassification of exposure.	Autism spectrum disorders and various vaccines; IBD and MMR vaccine; GBS and H1N1 vaccine
Self-controlled case series (SCCS)/self-controlled risk interval (SCRI)	Comparison between incidence rates in exposed time periods vs. incidence rates of self-matched unexposed time periods; SCCS: cases only; SCRI: vaccinated persons (only cases informative).	Adjust for time-invariant confounders; SCCS: Assess multiple occurrences of independent events within an individual; SCRI: Less susceptible to misclassification of exposure.	Time-varying confounding; Reverse causality bias.	GBS and H1N1 vaccine; Autism spectrum disorders and various vaccines; Seizures and various vaccines.

#### Cohort Studies

In observational cohort studies, there are two main temporal choices of comparator: historical or concurrent. A historical comparison uses data from previous studies to compute expected rates to compare to the observed rates of AEFI in the current vaccination situation (Belongia et al., 2010); while a concurrent cohort design follows groups of vaccinated and unvaccinated individuals forward in time and compares the frequency of the event (i.e., incidence rates, incidence rate ratio, hazard ratios, risk ratios etc).

Alleged strengths of the historical comparator design include greater statistical power to detect rare AEFIs due to a stable comparator based on large sample size, as well as improved timeliness in detecting potential safety signals by leveraging retrospective data for analysis. However, there are several limitations (Mesfin et al., 2019). First, the historical population must be similar to the vaccinated one in terms of baseline risk. Second, the design is subject to temporal confounders, such as seasonality, changing trends in the detection of AEFIs, and variation in diagnostic or coding criteria over time. These kinds of biases are of particular concern in COVID-19 vaccination surveillance since the frequency of patient visits before the pandemic differs greatly from during the pandemic when restrictions were in place. In addition, the transmission of other infectious diseases are also less likely to occur due to precautionary measures such as mask wearing, social distancing and frequent hand washing.

Various clinical projects have applied historical rate comparisons, including the CDC’s VSD project, which used background rates to detect safety signals for the adult tetanus-diphtheria-acellular pertussis (Tdap) vaccine (Yih et al., 2009), the human papillomavirus vaccine (HPV) (Black et al., 2009; Wijnans et al., 2013; Barker and Snape, 2014), and a broad range of pediatric vaccines (Lieu et al., 2007; Yih et al., 2011). Historical data were used in Europe to detect signals for the influenza A H1N1 vaccine (Black et al., 2009; Wijnans et al., 2013; Barker and Snape, 2014) and in Australia to detect signals for the rotavirus vaccine (Buttery et al., 2011).

An alternative is using the concurrent cohort design. For non-recurrent events (e.g., sudden infant death syndrome), the person-time at risk in cases ends with the events; while for recurrent events (e.g., febrile convulsions), the entire observation period is included in the person-time denominators (Farrington et al., 1995; Whitaker et al., 2009). There are, however, some caveats—the study design requires a sufficiently sized control group, which may be challenging to obtain when high vaccine coverage rates are expected. In addition, as with the historical comparator design, the vaccinated and unvaccinated populations are often likely to differ in terms of socioeconomic status, ethnicity and comorbidities, which may induce bias. In settings with limited resources (e.g., low-income countries), data on potential confounding variables are also often either not collected, or unavailable.

Some examples of concurrent cohort studies in VSS include a recent study in Western Australia looking at the association between seasonal influenza vaccination and AEFI (Salter et al., 2021) and also the CDC’s VSD project, which used a combination of historic and concurrent cohort study designs to study the association between HPV4 and AEFI among young women (Gee et al., 2011).

#### Case-Control Studies

In a case-control study, a group of cases from the source population is compared to a control group of event-free individuals representing the same source population from the same time-period. Controls are often matched to the cases on one or more variables at the date of the event, which requires accounting for this matching at the analysis stage. This design is best for rare events when data needs to be collected, as it only utilizes a small sample of data from the entire cohort (i.e., resource-efficient). While this study design can be economical for rare events, especially when specific data collection for included study individuals is required, identifying an appropriate control group is a potential limiting factor (Baker et al., 2015). When all data has already been collected (e.g., in secondary use of existing healthcare data), there is limited benefit to using case-control over other designs (Schuemie et al., 2019).

Case-control studies have been used to study the relationship between autism spectrum disorders and vaccines (Taylor et al., 2014), inflammatory bowel disease and measles-mumps-rubella (MMR) (Davis et al., 2001); pervasive development disorder and MMR (Smeeth et al., 2004), as well as Guillain-Barre syndrome and influenza A (H1N1) vaccine (Dieleman et al., 2011).

#### Self-Controlled Designs

##### Self-Controlled Case Series

The SCCS is a relatively newer study design used to estimate the relative incidence of rare adverse events after vaccination (Farrington et al., 1995; Whitaker et al., 2009). In this study design, incidence rates during exposed time are compared to incidence rates during unexposed time, but only cases are included, thus avoiding the need for large population cohorts or the need for selecting controls. Each case acts as its own control, thereby adjusting for both measured and unmeasured confounding variables that do not vary appreciably over time. Another advantage of the SCCS is that multiple occurrences of independent events within an individual can be used to inform the analysis. It is reported to be as powerful as a full cohort analysis, as non-cases would contribute very little information about the vaccine effect (Farrington, 2004). SCCS can be implemented efficiently using data from readily available sources. Access to quality data (i.e., preferably computerised vaccination records that can be linked to cases and ascertainment of cases independent of vaccination status), is crucial to optimise the use of this study design (Farrington, 2004).

##### Self-Controlled Risk Interval

Another alternative is the self-controlled risk interval (SCRI) method. This design includes vaccinated individuals only and compares the incidence rates during risk and non-risk timeframes, using only one nominated unexposed risk interval, defined relative to the time of vaccination (e.g., the period 30–1 day before vaccination, or the period 42–60 days after vaccination). The risk interval is the time period immediately following vaccination, and events that occur during this time-frame are categorized as exposed events (Salmon et al., 2013; Grave et al., 2020). This design is ideal for assessing the risk of any acute, self-limiting events following vaccination, but works less well for events that do not clearly and rapidly resolve.

While use of both the SCCS and SCRI designs does minimize selection bias as only the vaccinated individuals are studied, the trade-off is that the risk inferences are only applicable to the vaccinated population.

The SCCS and SCRI designs have been used extensively in VSS for influenza, MMR, and vaccines containing pertussis antigens. They have also been applied across a wide range of adverse events, including, but not limited to purpuras (Miller et al., 2001; France et al., 2008; Stowe et al., 2008), autisms (Taylor et al., 1999; Farrington et al., 2001; Andrews et al., 2002), seizures (Huang et al., 2010; Klein et al., 2010), meningitis (Dourado et al., 2000; Miller et al., 2007), asthmas (Kramarz et al., 2000; Kramarz et al., 2001), Guillain-Barre syndrome (Salmon et al., 2013; Grave et al., 2020) etc.

One limitation across the types of common epidemiological designs listed above is that there is often variation in vaccine data availability, as well as complexities in data access and data linkage requirements (Duszynski et al., 2021). Generally, there is no one best study design that is superior over the other. The most appropriate study design will depend on the specifics of the particular situation such as availability of resources, access to records (including how exposure and outcomes are reported), the number and distribution of cases and availability of population coverage data.

## Types of Common Biases and Confounders

Several assumptions have to be made while using the various study designs, and while the goal is to mitigate any biases as much as possible, violations of these assumptions are often still present to varying degrees.

### Selection Bias

One of the assumptions is that there is equal susceptibility among all individuals in the population to the disease (i.e., COVID-19). This may not always be true, as the level of natural immunity or susceptibility to the disease (i.e., COVID-19) may differ between those vaccinated and non-vaccinated. Another possibility of selection bias is related to the way sampling is done. If the individuals in whom the vaccine-adverse event association has been analysed differ from the source population in ways linked to both exposure to the vaccine and development of the adverse event, the resulting estimate of association will be biased (Institute of Medicine US et al., 1994). This is of special concern in the case-control design, as incorrect sampling may result in a non-intended biased vaccine coverage (i.e., not representing the source population). Selection bias may be avoided by sampling controls in a manner to ensure that they represent the exposure distribution (i.e., vaccine coverage) in the population.

### Misclassification Bias

#### Exposure Misclassification Bias

Exposure misclassification bias may occur if the vaccine exposure is not well recorded, leading to a vaccinated person being classified as unvaccinated or vice versa. This bias may also occur in the absence of a robust linkage of records for vaccination status. Generally, differential exposure misclassification (i.e., differential with respect to outcome status) is unlikely since exposure assessment (registration of vaccination) generally precedes the outcome and is unaffected by it. Problems will only arise if outcomes admitted to hospital are differentially registered and picked up as vaccinated in some healthcare settings. This type of bias however, may be problematic in designs using a contemporary comparator (e.g., contemporary cohort design and the case-control design), as individuals listed as “unexposed” in these study designs maybe vaccinated, thus biasing the results towards the null.

#### Outcome Misclassification Bias

In studies of vaccine adverse events, presumptive outcomes are often identified within a short period around the vaccination timeframe. Misclassification of these presumptive outcomes may occur due to miscoding or rule-out diagnoses (Newcomer et al., 2018), of which no adverse events were presumed to occur in the absence of further information. One way to mitigate this is to review all presumed outcomes and then re-analyse data with only confirmed outcomes (McNeil et al., 2014). Monitoring chronic vaccine adverse events poses more challenges for addressing misclassification bias as observation time may span years; and it would not be feasible to adjudicate the large number of presumptive outcomes identified in the data sources (Glanz et al., 2016).

Another potential source of outcome misclassification bias is diagnostic bias, which occurs when a specific adverse event is hypothesized and publicized to be linked to a vaccine, leading to preferentially ascertained cases due to awareness (Rodrigues and Smith, 1999). Another possibility is that those who have been vaccinated may be more likely to report possible AEFIs to their healthcare providers when they occur, as compared to the unvaccinated population. While the following is recommended for all study designs, it is of particular importance here, to use cases diagnosed in an already established information system before suspicion of the link was raised, and that the diagnoses of adverse event were done independent of vaccination status. If the study must be concurrent, cases should be sought within an established dataset to ensure that ascertainment bias is minimized, even if diagnosis bias is not completely avoided (Rodrigues and Smith, 1999).

Generally, misclassification bias is common in cohort and case-control study designs, but less common in the SCCS (when restricting to the vaccinated only population), and SCRI study designs (Baker et al., 2015).

### Confounding

#### Confounding by Indication

Another common type of bias is confounding by indication. While one might think that the probability of being vaccinated is independent from the probability of developing the outcome (i.e., COVID-19), it is often not the case. Individuals who are more likely to develop COVID-19 or at higher risk of severe infection (i.e., older, or with underlying comorbidities) are often prioritized in vaccination programs (Public Health England, 2021). To reduce confounding by indication, various design and analysis methods are used. Design approaches that have been proposed include comparing groups with similar prognosis (e.g., use of historical controls), or restricting the study population on levels of important confounding variables such as age and sex (Hak et al., 2002). Confounding by indication may also be mitigated by using a multivariable regression model or propensity score adjustments (Hak et al., 2002).

#### Time-Varying Confounding

Time-varying confounding occurs when confounders change over time. It often occurs with time-varying variables such as age, seasonality, and in the context of this pandemic - the emergence of new variants and rapid policy changes in vaccination programmes. These changes can be particularly tricky for cohort and case-control studies as conditions change during the study period. Time-varying confounding is also of particular concern in self-controlled study designs, especially if seasonal effects were not accounted for (Glanz et al., 2006). Thus, it has been proposed that any time-varying confounders should be explicitly defined and added to multiple Poisson regression models (Farrington et al., 1996). One of the challenge however, is that the form of seasonal variable may be difficult to be explicitly defined prior to conducting the analysis when the event is rare due to insufficient information to estimate the seasonal effect. This can be mitigated by using data of unexposed cases to fit the seasonal effect, or using splines for flexibility and regularization for robustness when power is low, which can be implemented using the OHDSI SCCS software. The SCRI method allows minimization of the effects of time-varying confounders by restricting the control (unexposed) time to a small time-window close to the time of vaccination.

## Prior Comparisons of Study Designs and Statistical Methods

Few systematic comparisons have been made of important methods for VSS. McClure et al. performed a simulation study to compare the above-mentioned four study designs in the context of VSS, comparing the following parameters: 1) detection time; 2) empirical power; 3) empirical false positive error rate, and 4) risk estimate bias (McClure et al., 2008). Detection time was defined as the first weekly interval where the log-likelihood ratio (LLR) exceeded the pre-specified upper bound (using Maximized Sequential Probability Ratio Test - MaxSPRT), corrected for sequential analysis, in at least 80% of iterations per design, vaccine pattern and event rate (McClure et al., 2008). Detection time was shortest in the matched cohort design, followed by the SCRI, SCCS, and case–control study design. In their simulation study, the minimum acceptable empirical power of the LLR was set at 80%, using the MaxSPRT. The risk-interval design used half of the subjects used by the cohort design, and its empirical power was generally within 2% of the cohort design. The SCCS design, requiring less data for stable estimates, still produced results within 3% of the cohort design. The authors also reported that the case–control design consistently underperformed relative to the other designs. An empirical false positive error rate for each study design was also calculated, defined as the percentage of simulated LLRs that exceeded the critical value upper bound when the true relative rate was null. The authors reported that for the majority of weeks for any of the study designs, the error rate was measured as zero, i.e., <1%. Lastly, the risk estimate bias was calculated as the percent difference of the regression estimate and the true estimate. For any of the study designs, the mean risk estimate decreased as monitoring time increased. The risk estimate bias was smallest for the cohort, followed by the risk-interval and SCCS designs, with the largest for the case–control design in most monitoring weeks, incidences and relative risk levels. This should however, be interpreted with caution as the effects of misclassification or confounding were not included in this simulation study.

The same authors later performed a follow-up study, with the major difference being the simulation of one type of unmeasured confounding (i.e., seasonality) in the latter (Glanz et al., 2006). Using 250 case sets of simulated data, the authors constructed three study designs (compared with the cohort study design) at three different incident rate ratios with decreasing disease incidence and simulated two confounding levels for both the fixed and seasonal confounding (Glanz et al., 2006). Regression analysis was used to compare the design-specific beta-estimates across study designs. The authors concluded that when compared to the cohort study design, the case-control design had lowest empirical power, highest mean standard errors and highest mean percent bias in the presence of fixed confounding, but when seasonal effect was incorporated as a time-varying confounder, the biased estimates were largely minimized. The SCCS and risk-interval designs, on the other hand, were comparable to the cohort design and demonstrated the ability to control for fixed confounding. The mean percent biases for these designs were, however, higher than those of the case-control when seasonality was not accounted for. There were, however, some limitations, including 1) use of simulated data, which may not be a true reflection of real-life scenario; and 2) the simplicity of the simulation using only one vaccination pattern (i.e., MMR) and one time-varying confounding variable (i.e., seasonality). Incorporation of various time-varying factors (e.g., health status and vaccination patterns), as well as adjusting for various biases and confounders is necessarily to provide more robust results.

In the following section, we provide the rationale of the EUMAEUS study, and how the use of real-world multi-database networks can help bridge the limitations of the above studies and provide more insights, not only from a methodological perspective, but also from a clinical perspective of vaccine safety surveillance.

## Rationale of the EUMAEUS Study–Importance of Using Real-World Multi-Database Networks for Methods Evaluation and Vaccine Safety Surveillance

### Methods Evaluation

In the previous section, we discussed how comparison of various performance metrics across various study designs were carried out using simulated data. The question of how well these metrics perform in real-world data, however, is largely unexplored. Therefore, a large-scale empirical evaluation comparing the various analytical methods, using various types of real-world data (e.g., claims and EHRs) is important. The EUMAEUS project within the OHDSI network provides an excellent platform to address this. One major strength of this project is the use of real negative controls, which allows evaluating the time to detection in a timely manner. Other advantages include the ability to explore the heterogeneity of vaccine uptake across databases, which allows the effect of different vaccine types or specific patterns of utilization to be examined (European Network of Centres for Pharmacoepidemiology and Pharmacovigilance (ENCePP), 2021). In addition, the relatively large sample size also allows the exploration and comparison of many variants of study designs commonly used in VSS; as well as the ability to incorporate advanced statistical methods such as the use of splines for age and seasonality adjustments for SCCS, or propensity scores for the comparative cohort design.

### Vaccine Safety Surveillance

In addition to the added value of using real-world multi-database networks such as the OHDSI network for methodological evaluation, these networks are also useful for VSS. The relatively large sample size, as compared to use of spontaneous reports, is of extreme importance when identifying rare AEFI. Another major advantage is the ability to pool data or results, which provides insight into the generalisability of findings. In addition, it is also useful for long-term surveillance of vaccine safety at a large-scale population level. Another added advantage is the involvement of experts from various countries to address issues relating to case definitions, coding in databases and research practices to increase consistency of results across the databases.

### Challenges and Limitations of Real-World Multi-Database Networks

There are however, some challenges associated with the use of a multi-country multi-database networks such as OHDSI. The “observational real-world” aspect of the data present challenges often not present in simulated data. First, accrual of participants is often unpredictable and may vary in rate and population composition over time, depending on availability and supply of vaccines, as well as policy implementations, etc. Second, it is also prone to confounding (some of which may vary over time) and misclassifications, especially if the AEFI is rare (Nelson et al., 2012). Other challenges include differences in health care systems and type of vaccines selection; differences in the mechanisms of how data were collected and generated; differences in the type and quality of each database (i.e., primary care data, claims data); as well as differences in the ethical and governance requirements in each country on anonymization of data and data sharing (European Network of Centres for Pharmacoepidemiology and Pharmacovigilance (ENCePP), 2021). While some of these challenges are inevitable, many may be overcome or alleviated by full commitment and good communication between data partners, good governance practices as well as maintaining an open-source network to ensure transparency and accountability.

### The EUMAEUS Study

As discussed in the previous sections, use of a multi-country multi-database network such as the OHDSI network is extremely valuable for method evaluation and VSS. In this section, we will present a brief overview of the EUMAEUS study, which aims to systematically evaluate the performance of methods (bias, precision and timeliness) across various study designs to reliably identify vaccine safety signals in real-world settings, to support efficient safety surveillance for COVID-19 vaccines.

#### Exposures, Outcomes and Data Sources

To evaluate the performance of method for VSS, we select prior vaccination for which we already have ample data available. The exposures of interest in EUMAEUS include six groups of vaccines, including A (H1N1)pdm09, seasonal flu (Fluvirin), seasonal flu (Fluzone), seasonal flu (All), zoster (Shingrix), and human papillomavirus (Gardasil 9), each with specific study periods. In terms of outcome, we will study the association between vaccinations and 1) outcomes believed to be unrelated to any of the vaccines with a similar prevalence and severity to the suspected AEFI (i.e., negative control outcome), and 2) outcomes simulated to be caused by the vaccines (i.e., imputed positive controls) to evaluate method performance. EUMAEUS will be executed as part of the OHDSI network study, of which we will be using a combination of administrative claims and EHR databases from the US. The use of all US-based databases may limit its generalizability, but an advantage would be less variation in policies and practices. Details of the databases can be found in our online protocol (Schuemie, 2021).

#### Methods Evaluation and Performance Metrics

We proposed four components for VSS, including 1) construction of a counterfactual (i.e., expected count), 2) a time-at-risk (TAR) when AEFI may occur; 3) appropriate test statistic to estimate the difference between observed vs. expected counts, and 4) a decision rule to classify true safety signals from non-signals.

For the counterfactual construction, we will be applying a total of 17 variations (based on evidence in literature) across four study designs, namely 1) cohort using a contemporary non-user comparator, 2) historic rates, 3) case-control, and 4) self-controlled case series (SCCS)/self-controlled risk interval (SCRI). For example, one of the variations to be used in the cohort method is anchoring the comparator on a random outpatient visit using 1-on-1 propensity score matching.

The TAR is the time window relative to the vaccination date, when outcomes are potentially attributed to the vaccine. TAR windows will be constructed for both the first and second dose, using three time-frames: 1–28 days, 1–42 days, and 0–1 day after vaccination.

To estimate the difference between observed vs expected counts, we will use the effect-size estimate (e.g., incidence rate ratio, hazard ratio or odds ratio) and log likelihood ratio.

To differentiate true safety signals from non-signals, we used the decision rule of applying the critical value for the LLR computed at an alpha of 0.05 using MaxSPRT. The Poisson model will be applied for the historical rate study design, and the binomial model for all other methods.

**FIGURE 1:**
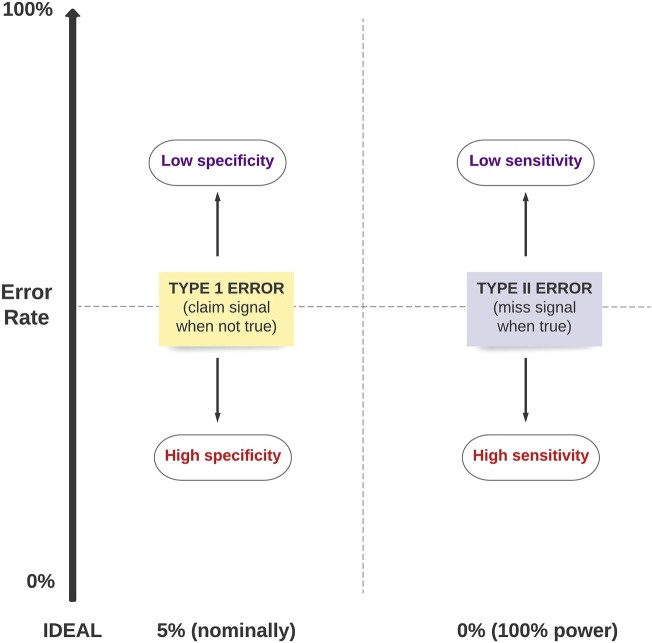
The relationship between Type 1 error, Type II error, sensitivity and specificity of a test.

We will be performing a range of performance metrics to separate true signals from non-signals across the range of four study designs mentioned above. Some of the decisions will be based on Type I or Type II error rates, which will in turn affect the sensitivity and specificity of the test. The relationship between Type I error, Type II error, sensitivity and specificity is summarized in Figure 1.

In addition, we will also be comparing the amount of time to achieve significant power to identify a true signal using the different study designs, defined as timeliness. The study period for each vaccine of interest will be divided into calendar months, and the performance metrics will be reported for each month.

Further details of the overall study design may be found on our online protocol (Schuemie, 2021).

## Conclusion

With the rapid, global COVID-19 vaccine rollout, it is highly likely that potential safety signals will emerge. It is therefore crucial to have a VSS system in place to facilitate early detection, investigation and analysis of any AEFI. Whilst there are many study designs and statistical methods available, each present different methodological challenges and are often affected by different types of biases, some of which may be mitigated through study design aspects such as matching, stratification or data restriction, or statistical analysis methods. While there have been simulation studies done and theoretical arguments presented to evaluate the performance metrics of some statistical methods across some different study designs, less is known on how they perform in a real-world setting. Although “real-world” observational data are prone to biases, which need to be identified and addressed as best possible, they remain to date the most feasible method to evaluate and quantify vaccine-related effects as vaccines are approved and reach real populations. We have here provided a background review and discussion of the current state of knowledge regarding different methods for VSS and hope that the EUMAEUS project that we have described, and which is now ongoing will help to further address some of the remaining questions involving the how, what and when to reliably identify vaccine safety signals in real-world settings.

## References

[B1] Andrews N. Miller E. Taylor B. Lingam R. Simmons A. Stowe J. (2002). Recall Bias, MMR, and Autism. Arch. Dis. Child. 87 (6), 493–494. 10.1136/adc.87.6.493 12456546 PMC1755823

[B2] Baker M. A. Lieu T. A. Li L. Hua W. Qiang Y. Kawai A. T. (2015). A Vaccine Study Design Selection Framework for the Postlicensure Rapid Immunization Safety Monitoring Program. Am. J. Epidemiol. 181 (8), 608–618. 10.1093/aje/kwu322 25769306

[B3] Baker M. A. Nguyen M. Cole D. V. Lee G. M. Lieu T. A. (2013). Post-licensure Rapid Immunization Safety Monitoring Program (PRISM) Data Characterization. Vaccine 31, K98–K112. 10.1016/j.vaccine.2013.04.088 24331080

[B4] Ball P. (2021). The Lightning-Fast Quest for COVID Vaccines - and what it Means for Other Diseases. Nature 589, 16–18. 10.1038/d41586-020-03626-1 33340018

[B5] Barker C. I. Snape M. D. (2014). Pandemic Influenza A H1N1 Vaccines and Narcolepsy: Vaccine Safety Surveillance in Action. Lancet Infect. Dis. 14 (3), 227–238. 10.1016/S1473-3099(13)70238-X 24360892

[B6] Basta N. E. Moodie EMM on Behalf of the McGill University COVID19 Vaccine Tracker Team. COVID-19 Vaccine Development and Approvals Tracker. 2020 Available from: covid19.trackvaccines.org .

[B7] Belongia E. A. Irving S. A. Shui I. M. Kulldorff M. Lewis E. Yin R. (2010). Real-time Surveillance to Assess Risk of Intussusception and Other Adverse Events after Pentavalent, Bovine-Derived Rotavirus Vaccine. Pediatr. Infect. Dis. J. 29 (1), 1–5. 10.1097/INF.0b013e3181af8605 19907356

[B8] Black S. Eskola J. Siegrist C. A. Halsey N. MacDonald N. Law B. (2009). Importance of Background Rates of Disease in Assessment of Vaccine Safety during Mass Immunisation with Pandemic H1N1 Influenza Vaccines. Lancet 374 (9707), 2115–2122. 10.1016/S0140-6736(09)61877-8 19880172 PMC2861912

[B9] Buttery J. P. Danchin M. H. Lee K. J. Carlin J. B. McIntyre P. B. Elliott E. J. (2011). Intussusception Following Rotavirus Vaccine Administration: post-marketing Surveillance in the National Immunization Program in Australia. Vaccine 29 (16), 3061–3066. 10.1016/j.vaccine.2011.01.088 21316503

[B10] Centers for Disease Control and Prevention Cisa (2021). Clinical Immunization Safety Assessment (CISA) Project. Available from: https://www.cdc.gov/vaccinesafety/ensuringsafety/monitoring/cisa/index.html?CDC_AA_refVal=https%3A%2F%2Fwww.cdc.gov%2Fvaccinesafety%2Factivities%2FCISA.html .

[B11] Centers for Disease Control and Prevention Vaers (2021). Vaccine Adverse Event Reporting System (VAERS). Available from: https://www.cdc.gov/vaccinesafety/ensuringsafety/monitoring/vaers/index.html .

[B12] Centers for Disease Control and Prevention Vsd (2021). Vaccine Safety Datalink (VSD). Available from: https://www.cdc.gov/vaccinesafety/ensuringsafety/monitoring/vsd/index.html .

[B13] Davis R. L. Kramarz P. Bohlke K. Benson P. Thompson R. S. Mullooly J. (2001). Measles-Mumps-Rubella and Other Measles-Containing Vaccines Do Not Increase the Risk for Inflammatory Bowel Disease: A Case-Control Study from the Vaccine Safety Datalink Project. Arch. Pediatr. Adolesc. Med. 155 (3), 354–359. 10.1001/archpedi.155.3.354 11231801

[B14] Dieleman J. Romio S. Johansen K. Weibel D. Bonhoeffer J. Sturkenboom M. (2011). Guillain-Barre Syndrome and Adjuvanted Pandemic Influenza A (H1N1) 2009 Vaccine: Multinational Case-Control Study in Europe. BMJ 343, d3908. 10.1136/bmj.d3908 21750072 PMC3134565

[B15] Dourado I. Cunha S. Teixeira M. G. Farrington C. P. Melo A. Lucena R. (2000). Outbreak of Aseptic Meningitis Associated with Mass Vaccination with a Urabe-Containing Measles-Mumps-Rubella Vaccine: Implications for Immunization Programs. Am. J. Epidemiol. 151 (5), 524–530. 10.1093/oxfordjournals.aje.a010239 10707922

[B16] Duszynski K. M. Stark J. H. Cohet C. Huang W. T. Shin J. Y. Lai E. C. (2021). Suitability of Databases in the Asia-Pacific for Collaborative Monitoring of Vaccine Safety. Pharmacoepidemiol. Drug Saf. 30 (7), 843–857. 10.1002/pds.5214 33634545

[B17] European Medicine Agency Ema (2020b). Pharmacovigilance Plan of the EU Regulatory Network for COVID-19 Vaccines (EMA/333964/2020). Available from: https://www.ema.europa.eu/en/documents/other/pharmacovigilance-plan-eu-regulatory-network-covid-19-vaccines_en.pdf .

[B18] European Medicines Agency Ema (2021). COVID-19 Vaccines. Available from: https://www.ema.europa.eu/en/human-regulatory/overview/public-health-threats/coronavirus-disease-covid-19/treatments-vaccines/covid-19-vaccines .

[B19] European Medicines Agency Ema (2020a). Signal Assessment Report on Embolic and Thrombotic Events (SMQ) with COVID-19 Vaccine (ChAdOx1-S [recombinant]) – COVID-19 Vaccine AstraZeneca. Available from: https://www.ema.europa.eu/en/documents/prac-recommendation/signal-assessment-report-embolic-thrombotic-events-smq-covid-19-vaccine-chadox1-s-recombinant-covid_en.pdf .

[B20] European Network of Centres for Pharmacoepidemiology and Pharmacovigilance (ENCePP) (2021). Guide on Methodological Standards in Pharmacoepidemiology (Revision 9). EMA/95098/2010. Available from: http://www.encepp.eu/standards_and_guidances/methodologicalGuide.shtml .

[B21] Farrington C. P. (2004). Control without Separate Controls: Evaluation of Vaccine Safety Using Case-Only Methods. Vaccine 22 (15-16), 2064–2070. 10.1016/j.vaccine.2004.01.017 15121324

[B22] Farrington C. P. Miller E. Taylor B. (2001). MMR and Autism: Further Evidence against a Causal Association. Vaccine 19 (27), 3632–3635. 10.1016/s0264-410x(01)00097-4 11395196

[B23] Farrington C. P. Nash J. Miller E. (1996). Case Series Analysis of Adverse Reactions to Vaccines: a Comparative Evaluation. Am. J. Epidemiol. 143 (11), 1165–1173. 10.1093/oxfordjournals.aje.a008695 8633607

[B24] Farrington P. Pugh S. Colville A. Flower A. Nash J. Morgan-Capner P. (1995). A New Method for Active Surveillance of Adverse Events from Diphtheria/tetanus/pertussis and Measles/mumps/rubella Vaccines. Lancet 345 (8949), 567–569. 10.1016/s0140-6736(95)90471-9 7619183

[B25] France E. K. Glanz J. Xu S. Hambidge S. Yamasaki K. Black S. B. (2008). Risk of Immune Thrombocytopenic Purpura after Measles-Mumps-Rubella Immunization in Children. Pediatrics 121 (3), e687–92. 10.1542/peds.2007-1578 18310189

[B26] Gee J. Naleway A. Shui I. Baggs J. Yin R. Li R. (2011). Monitoring the Safety of Quadrivalent Human Papillomavirus Vaccine: Findings from the Vaccine Safety Datalink. Vaccine 29 (46), 8279–8284. 10.1016/j.vaccine.2011.08.106 21907257

[B27] Glanz J. M. McClure D. L. Xu S. Hambidge S. J. Lee M. Kolczak M. S. (2006). Four Different Study Designs to Evaluate Vaccine Safety Were Equally Validated with Contrasting Limitations. J. Clin. Epidemiol. 59 (8), 808–818. 10.1016/j.jclinepi.2005.11.012 16828674

[B28] Glanz J. M. Newcomer S. R. Jackson M. L. Omer S. B. Bednarczyk R. A. Shoup J. A. (2016). White Paper on Studying the Safety of the Childhood Immunization Schedule in the Vaccine Safety Datalink. Vaccine 34 (Suppl. 1), A1–a29. 10.1016/j.vaccine.2015.10.082 26830300

[B29] Grave C. Boucheron P. Rudant J. Mikaeloff Y. Tubert-Bitter P. Escolano S. (2020). Seasonal Influenza Vaccine and Guillain-Barré Syndrome: A Self-Controlled Case Series Study. Neurology 94 (20), e2168–e2179. 10.1212/WNL.0000000000009180 32098853

[B30] Hak E. Verheij T. J. Grobbee D. E. Nichol K. L. Hoes A. W. (2002). Confounding by Indication in Non-experimental Evaluation of Vaccine Effectiveness: the Example of Prevention of Influenza Complications. J. Epidemiol. Community Health 56 (12), 951–955. 10.1136/jech.56.12.951 12461118 PMC1756997

[B31] Holder J. (2021). Tracking Coronavirus Vaccinations Around the World. Available from: https://www.nytimes.com/interactive/2021/world/covid-vaccinations-tracker.html .

[B32] Huang W. T. Gargiullo P. M. Broder K. R. Weintraub E. S. Iskander J. K. Klein N. P. (2010). Lack of Association between Acellular Pertussis Vaccine and Seizures in Early Childhood. Pediatrics 126 (2), 263–269. 10.1542/peds.2009-1496 20643726

[B33] Institute of Medicine Us (1994). in Vaccine Safety Committee, *Adverse Events Associated with Childhood Vaccines: Evidence Bearing on Causality* . Editors Stratton C. J. H. K. R. JohnstonJr. R. B. (Washington (DC): National Academies Press US). 25144097

[B34] John Hopkins University (2021). University and Coronavirus Resource Center. Available from: https://coronavirus.jhu.edu/map.html .

[B35] Klein N. P. Fireman B. Yih W. K. Lewis E. Kulldorff M. Ray P. (2010). Measles-mumps-rubella-varicella Combination Vaccine and the Risk of Febrile Seizures. Pediatrics 126 (1), e1–8. 10.1542/peds.2010-0665 20587679

[B36] Kramarz P. Destefano F. Gargiullo P. M. Chen R. T. Lieu T. A. Davis R. L. (2001). Does Influenza Vaccination Prevent Asthma Exacerbations in Children? J. Pediatr. 138 (3), 306–310. 10.1067/mpd.2001.112168 11241034

[B37] Kramarz P. DeStefano F. Gargiullo P. M. Davis R. L. Chen R. T. Mullooly J. P. (2000). Does Influenza Vaccination Exacerbate Asthma? Analysis of a Large Cohort of Children with Asthma. Vaccine Safety Datalink Team. Arch. Fam. Med. 9 (7), 617–623. 10.1001/archfami.9.7.617 10910309

[B38] Lane J. C. E. Weaver J. Kostka K. Duarte-Salles T. Abrahao M. T. F. Alghoul H. (2020). Risk of Hydroxychloroquine Alone and in Combination with Azithromycin in the Treatment of Rheumatoid Arthritis: a Multinational, Retrospective Study. Lancet Rheumatol. 2 (11), e698–e711. 10.1016/S2665-9913(20)30276-9 32864627 PMC7442425

[B39] Leite A. Andrews N. J. Thomas S. L. (2016). Near Real-Time Vaccine Safety Surveillance Using Electronic Health Records-A Systematic Review of the Application of Statistical Methods. Pharmacoepidemiol. Drug Saf. 25 (3), 225–237. 10.1002/pds.3966 26817940 PMC5021108

[B40] Lieu T. A. Kulldorff M. Davis R. L. Lewis E. M. Weintraub E. Yih K. (2007). Real-time Vaccine Safety Surveillance for the Early Detection of Adverse Events. Med. Care 45 (10 Suppl. 2), S89–S95. 10.1097/MLR.0b013e3180616c0a 17909389

[B41] McClure D. L. Glanz J. M. Xu S. Hambidge S. J. Mullooly J. P. Baggs J. (2008). Comparison of Epidemiologic Methods for Active Surveillance of Vaccine Safety. Vaccine 26 (26), 3341–3345. 10.1016/j.vaccine.2008.03.074 18462849

[B42] McNeil M. M. Gee J. Weintraub E. S. Belongia E. A. Lee G. M. Glanz J. M. (2014). The Vaccine Safety Datalink: Successes and Challenges Monitoring Vaccine Safety. Vaccine 32 (42), 5390–5398. 10.1016/j.vaccine.2014.07.073 25108215 PMC6727851

[B43] Mesfin Y. M. Cheng A. Lawrie J. Buttery J. (2019). Use of Routinely Collected Electronic Healthcare Data for Postlicensure Vaccine Safety Signal Detection: a Systematic Review. BMJ Glob. Health 4 (4), e001065. 10.1136/bmjgh-2018-001065 PMC6615875 31354969

[B44] Miller E. Andrews N. Stowe J. Grant A. Waight P. Taylor B. (2007). Risks of Convulsion and Aseptic Meningitis Following Measles-Mumps-Rubella Vaccination in the United Kingdom. Am. J. Epidemiol. 165 (6), 704–709. 10.1093/aje/kwk045 17204517

[B45] Miller E. Waight P. Farrington C. P. Andrews N. Stowe J. Taylor B. (2001). Idiopathic Thrombocytopenic Purpura and MMR Vaccine. Arch. Dis. Child. 84 (3), 227–229. 10.1136/adc.84.3.227 11207170 PMC1718684

[B46] Morales D. R. Conover M. M. You S. C. Pratt N. Kostka K. Duarte-Salles T. (2021). Renin-angiotensin System Blockers and Susceptibility to COVID-19: an International, Open Science, Cohort Analysis. Lancet Digit Health 3 (2), e98–e114. 10.1016/S2589-7500(20)30289-2 33342753 PMC7834915

[B47] Moro P. L. Li R. Haber P. Weintraub E. Cano M. (2016). Surveillance Systems and Methods for Monitoring the post-marketing Safety of Influenza Vaccines at the Centers for Disease Control and Prevention. Expert Opin. Drug Saf. 15 (9), 1175–1183. 10.1080/14740338.2016.1194823 27268157 PMC6500454

[B48] Nelson J. C. Cook A. J. Yu O. Dominguez C. Zhao S. Greene S. K. (2012). Challenges in the Design and Analysis of Sequentially Monitored Postmarket Safety Surveillance Evaluations Using Electronic Observational Health Care Data. Pharmacoepidemiol. Drug Saf. 21 (Suppl. 1), 62–71. 10.1002/pds.2324 22262594

[B49] Newcomer S. R. Kulldorff M. Xu S. Daley M. F. Fireman B. Lewis E. (2018). Bias from Outcome Misclassification in Immunization Schedule Safety Research. Pharmacoepidemiol. Drug Saf. 27 (2), 221–228. 10.1002/pds.4374 29292551 PMC5800415

[B50] Observational Health Data Sciences and Informatics (Ohdsi) (2021). COVID-19 Updates Page. Available from: https://www.ohdsi.org/covid-19-updates/ .

[B51] Prats-Uribe A. Sena A. G. Lai L. Y. H. Ahmed W. U. Alghoul H. Alser O. (2021). Use of Repurposed and Adjuvant Drugs in Hospital Patients with Covid-19: Multinational Network Cohort Study. Bmj 373, n1038. 10.1136/bmj.n1038 33975825 PMC8111167

[B52] Public Health England (2021). COVID-19 Vaccination in the UK: a Guide to Phase 2 of the Programme 2021 14. Available from: https://www.gov.uk/government/publications/covid-19-vaccination-guide-for-older-adults/covid-19-vaccination-a-guide-to-phase-2-of-the-programme .

[B53] Rodrigues L. C. Smith P. G. (1999). Use of the Case-Control Approach in Vaccine Evaluation: Efficacy and Adverse Effects. Epidemiol. Rev. 21 (1), 56–72. 10.1093/oxfordjournals.epirev.a017988 10520473

[B54] Salmon D. A. Proschan M. Forshee R. Gargiullo P. Bleser W. Burwen D. R. (2013). Association between Guillain-Barré Syndrome and Influenza A (H1N1) 2009 Monovalent Inactivated Vaccines in the USA: a Meta-Analysis. Lancet 381 (9876), 1461–1468. 10.1016/S0140-6736(12)62189-8 23498095

[B55] Salter S. Singh G. Nissen L. Trentino K. Murray K. Lee K. (2021). Active Vaccine Safety Surveillance of Seasonal Influenza Vaccination via a Scalable, Integrated System in Western Australian Pharmacies: a Prospective Cohort Study. BMJ Open 11 (6), e048109. 10.1136/bmjopen-2020-048109 PMC8190048 34103321

[B56] Schuemie M. J. (2021). Research Protocol: EUMAEUS: Evaluating Use of Methods for Adverse Event under Surveillance (For Vaccines) [Version 1.2.0]. Available from: https://ohdsi-studies.github.io/Eumaeus/Protocol.html#6_Rationale_and_Background .

[B57] Schuemie M. J. Ryan P. B. Man K. K. C. Wong I. C. K. Suchard M. A. Hripcsak G. (2019). A Plea to Stop Using the Case-Control Design in Retrospective Database Studies. Stat. Med. 38 (22), 4199–4208. 10.1002/sim.8215 31436848 PMC6771795

[B58] Smeeth L. Cook C. Fombonne E. Heavey L. Rodrigues L. C. Smith P. G. (2004). MMR Vaccination and Pervasive Developmental Disorders: a Case-Control Study. Lancet 364 (9438), 963–969. 10.1016/S0140-6736(04)17020-7 15364187

[B59] Stowe J. Kafatos G. Andrews N. Miller E. (2008). Idiopathic Thrombocytopenic Purpura and the Second Dose of MMR. Arch. Dis. Child. 93 (2), 182–183. 10.1136/adc.2007.126003 17962371

[B60] Taylor B. Miller E. Farrington C. P. Petropoulos M. C. Favot-Mayaud I. Li J. (1999). Autism and Measles, Mumps, and Rubella Vaccine: No Epidemiological Evidence for a Causal Association. Lancet 353 (9169), 2026–2029. 10.1016/s0140-6736(99)01239-8 10376617

[B61] Taylor L. E. Swerdfeger A. L. Eslick G. D. (2014). Vaccines Are Not Associated with Autism: an Evidence-Based Meta-Analysis of Case-Control and Cohort Studies. Vaccine 32 (29), 3623–3629. 10.1016/j.vaccine.2014.04.085 24814559

[B62] Uk Medicines and Healthcare products Regulatory Agency (2021). Coronavirus Vaccine - Weekly Summary of Yellow Care Reporting. Available from: https://www.gov.uk/government/publications/coronavirus-covid-19-vaccine-adverse-reactions/coronavirus-vaccine-summary-of-yellow-card-reporting .

[B63] Us Food & Drug Administration (Fda) (2021). COVID-19 Vaccines. Available from: https://www.fda.gov/emergency-preparedness-and-response/coronavirus-disease-2019-covid-19/covid-19-vaccines .

[B64] Whitaker H. J. Hocine M. N. Farrington C. P. (2009). The Methodology of Self-Controlled Case Series Studies. Stat. Methods Med. Res. 18 (1), 7–26. 10.1177/0962280208092342 18562396

[B65] Wijnans L. Lecomte C. de Vries C. Weibel D. Sammon C. Hviid A. (2013). The Incidence of Narcolepsy in Europe: before, during, and after the Influenza A(H1N1)pdm09 Pandemic and Vaccination Campaigns. Vaccine 31 (8), 1246–1254. 10.1016/j.vaccine.2012.12.015 23246544

[B66] World Health Organization (Who) (2021). Establishing Surveillance Systems in Countries Using COVID-19 Vaccines. [cited 2022 January 29, 2022].

[B67] Yih W. K. Kulldorff M. Fireman B. H. Shui I. M. Lewis E. M. Klein N. P. (2011). Active Surveillance for Adverse Events: the Experience of the Vaccine Safety Datalink Project. Pediatrics 127 (Suppl. 1), S54–S64. 10.1542/peds.2010-1722I 21502252

[B68] Yih W. K. Nordin J. D. Kulldorff M. Lewis E. Lieu T. A. Shi P. (2009). An Assessment of the Safety of Adolescent and Adult Tetanus-Diphtheria-Acellular Pertussis (Tdap) Vaccine, Using Active Surveillance for Adverse Events in the Vaccine Safety Datalink. Vaccine 27 (32), 4257–4262. 10.1016/j.vaccine.2009.05.036 19486957

